# Methylation analysis and diagnostics of Beckwith-Wiedemann syndrome in 1,000 subjects

**DOI:** 10.1186/1868-7083-6-11

**Published:** 2014-06-04

**Authors:** Abdulla Ibrahim, Gail Kirby, Carol Hardy, Renuka P Dias, Louise Tee, Derek Lim, Jonathan Berg, Fiona MacDonald, Peter Nightingale, Eamonn R Maher

**Affiliations:** 1 Department of Medical Genetics, University of Cambridge and NIHR Cambridge Biomedical Research Centre, Cambridge CB2 0QQ, UK; 2 Department of Clinical Genetics, University of Dundee, Ninewells Hospital and Medical School, Dundee DD1 9SY, UK; 3 Centre for Rare Diseases and Personalised Medicine, School of Clinical and Experimental Medicine, College of Medical and Dental Sciences, University of Birmingham, Birmingham B15 2TT, UK; 4 West Midlands Regional Genetics Service, Birmingham Women’s Hospital, Birmingham B15 2TG, UK; 5 Wellcome Trust Clinical Research Facility, University Hospitals Birmingham NHS Foundation Trust, Queen Elizabeth Hospital, Birmingham B15 2TH, UK

**Keywords:** Beckwith-Wiedemann syndrome, Imprinting, 11p15, Diagnostic criteria, Scoring system

## Abstract

**Background:**

Beckwith-Wiedemann syndrome (BWS), a congenital overgrowth disorder with variable expressivity and a predisposition to tumorigenesis, results from disordered expression and/or function of imprinted genes at chromosome 11p15.5. There are no generally agreed clinical diagnostic criteria, with molecular studies commonly performed to confirm diagnosis. In particular, methylation status analysis at two 11p15.5 imprinting control centres (IC1 and IC2) detects up to 80% of BWS cases (though low-level mosaicism may not be detected). In order to evaluate the relationship between the clinical presentation of suspected BWS and IC1/2 methylation abnormalities we reviewed the results of >1,000 referrals for molecular diagnostic testing.

**Results:**

Out of 1,091 referrals, 507 (46.5%) had a positive diagnostic test for BWS. The frequency of tumours was 3.4% in those with a molecular diagnosis of BWS. Previously reported genotype-phenotype associations with paternal uniparental disomy, IC1, and IC2 epimutation groups were confirmed and potential novel associations detected. Predictive values of previously described clinical diagnostic criteria were compared and, although there were differences in their sensitivity and specificity, receiver operating characteristic (ROC) analysis demonstrated that these were not optimal in predicting 11p15.5 methylation abnormalities. Using logistic regression, we identified clinical features with the best predictive value for a positive methylation abnormality. Furthermore, we developed a weighted scoring system (sensitivity 75.9%, and specificity 81.8%) to prioritise patients presenting with the most common features of BWS, and ROC analysis demonstrated superior performance (area under the curve 0.85, 95% CI 0.83 to 0.87) compared to previous criteria.

**Conclusions:**

We suggest that this novel tool will facilitate selection of patients with suspected BWS for routine diagnostic testing and so improve the diagnosis of the disorder.

## Background

Beckwith-Wiedemann syndrome (BWS; MIM #130650), a congenital overgrowth disorder with a predisposition toward tumorigenesis, results from abnormal expression/function of imprinted genes from the chromosome 11p15.5 imprinted gene cluster [1-4]. Only a minority of human genes (around 100) are imprinted (that is, epigenetically regulated such that one allele is preferentially expressed according to the parent-of-origin of the allele) but imprinted genes characteristically occur in clusters and, to date, appear to be preferentially implicated in prenatal growth and development [5,6]. Since the first clinical description of BWS 60 years ago [7,8] there have been considerable advances in defining the molecular basis of this disorder (see [1-4] and references within). It is now recognised that increased IGF2 expression and/or loss of expression or inactivation of *CDKN1C* account for most cases of this disorder. Disordered IGF2/CDKN1C function may be caused by multiple mechanisms, including paternal uniparental disomy (pUPD), cytogenetic abnormalities (such as paternally inherited duplications and maternally inherited balanced translocations/inversions), imprinting centre (IC) mutations/deletions and epimutations, and *CDKN1C* mutations (see [1-4] and references within). Despite the marked heterogeneity of possible epigenetic/genetic mechanisms, most cases of BWS are sporadic and result from pUPD or IC epimutations, and diagnostic investigation for suspected BWS typically involves methylation analysis at two differentially methylated regions (*H19/IGF2* intergenic DMR and KvDMR1) that are coincident with the distal and centromeric imprinting centres (IC1 and IC2, respectively) [6,9-11]. Thus, approximately 5 to 10% of children with BWS have a gain of methylation on the maternal IC1 allele (normally only the paternal allele is methylated), which is associated with biallelic expression of *IGF2* and silencing of *H19* expression (normally *IGF2* is monoallelically expressed from the paternal allele and *H19* is expressed from the maternal allele only). A further 50% of individuals with BWS have a loss of methylation at IC2 (KvDMR1); usually the paternal IC2 allele is unmethylated and the maternal allele is methylated, but in these cases both alleles are unmethylated [11,12]. Such IC2 epimutations are associated with loss of function of the CDKN1C growth suppressor. Thus, *CDKN1C* is imprinted and preferentially expressed from the maternal allele, and a loss of maternal allele methylation at IC2 is associated with loss of maternal allele *CDKN1C* expression and hence a marked reduction in CDKN1C growth suppressor activity [13]. Whilst the frequency of *CDKN1C* mutations is small (approximately 5%) in sporadic cases, mutations may be detected in about 50% of familial BWS cases [14,15].

Though a number of different schemes have been suggested for the clinical diagnosis of BWS [16-20], there are no generally agreed criteria. Recently, molecular genetic diagnostic techniques have assumed an important role in facilitating the diagnosis of BWS. In particular, methylation profiling at IC1 and IC2 enables identification of individuals with the most frequent causes of BWS (pUPD, IC1, and IC2 epimutations) [10]. However, low-level mosaicism, as is often the case in pUPD, may not be detected by some methods of methylation analysis [21]. Given the high risk of tumorigenesis in this pUPD subgroup (24% by age 5 years) [12], identifying these patients is important in order to better manage their condition. Here, we have evaluated the clinical and molecular findings of >1,000 patients with suspected BWS referred to a tertiary molecular genetics laboratory for IC1 and IC2 methylation profiling. We analysed (a) the clinical features of different BWS molecular subgroups, (b) the frequency of positive molecular results according to different proposed diagnostic criteria, and (c) which of the common clinical features were most predictive of an abnormal IC1/2 methylation profile.

## Results

### Clinical features of patients referred for diagnostic testing

A total of 1,091 individuals (male:female = 1.06:1) were referred for diagnostic testing. The most frequent indications for referral were: macroglossia (52.3%), anterior abdominal wall defects (44.8%; exomphalos in 22.8%, umbilical hernia in 21.5%, and diastasis recti in 5.4%), hemihypertrophy (41.4%), pre- or postnatal macrosomia (38.7%), ear creases/pits (36.4%), neonatal hypoglycaemia (29.8%), facial naevus flammeus (FNF, 24.1%), organomegaly (17.0%), polyhydramnios (10.1%), maxillary hypoplasia (9.6%), congenital heart defects (8.3%), and embryonal tumours (3.1%). The median number of clinical features at referral was three (range 1 to 13). Of those with a single clinical indication (22.8% of total referrals), the most frequent was hemihypertrophy (present in 65.5% of those with a single feature). In those with two or more clinical indications for referral, the most frequent combinations of features were macroglossia and ear creases/pits (37.9%).

### Molecular genetic results and relationship to clinical indications for referral

Overall, 507/1,091 (46.5%) individuals tested had an abnormal methylation profile at IC1 and/or IC2. Forty-seven (4.3%) had hypermethylation at IC1 only, 321 (29.4%) had isolated IC2 hypomethylation, and 135 (12.4%) had abnormal IC1 and IC2 methylation from pUPD. Of those with an aberrant IC1 methylation profile, three (0.3%) individuals had a copy number abnormality (all duplications), while of those who had an aberrant IC2 methylation profile, one (0.1%) individual had a copy number abnormality (deletion). Thus, among patients with a positive molecular diagnosis, 63.3% had evidence of an IC2 epimutation, 26.6% pUPD, 9.3% IC1, and 0.8% had a copy number abnormality (it should be noted that our testing was not sensitive to all BWS methylation abnormalities: diagnoses due to low-level mosaicism may be missed [21,22] and some cases with normal BWS methylation profiles may have *CDKN1C* mutations [10,15]).

In general, the greater the number of clinical features of BWS present at referral, the greater the likelihood of a positive molecular diagnosis. Thus, 79.3% of those with five or more relevant clinical findings had a positive diagnostic test, and 65.2%, 45.5%, 26.4% and 12.4% of those with four, three, two and one clinical indications, respectively. Of those with isolated hemihypertrophy and no other clinical features, 12.9% had a positive diagnostic test.

Individual clinical features associated with a greater than average frequency of a positive diagnostic test for methylation abnormalities were: FNF (73.1%), diastasis recti (72.4%), organomegaly (72.3%), macroglossia (72.2%), polyhydramnios (71.6%), and exomphalos (70.0%), whereas the presence of hemihypertrophy was associated with a lower frequency of a positive diagnostic test (38.2%) (Table 1).

**Table 1:** Distribution of individual Beckwith-Wiedemann syndrome clinical features according to molecular subtype

	**pUPD**	**IC1**	**IC2**	**Total**
Facial naevus flammeus	21.1% (40/190)	3.7% (7/190)	75.3% (143/190)	73.1% (190/260)
Diastasis recti	33.3% (14/42)	23.8% (10/42)	42.9% (18/42)	72.4% (42/58)
Organomegaly	38.3% (51/133)	16.5% (22/133)	45.1% (60/133)	72.3% (133/184)
Macroglossia	22.5% (92/408)	8.1% (33/408)	69.4% (283/408)	72.2% (408/565)
Polyhydramnios	24.4% (19/78)	3.8% (3/78)	71.8% (56/78)	71.6% (78/109)
Exomphalos	6.9% (12/173)	1.7% (3/173)	91.3% (158/173)	70.0% (173/247)
Prognathism	22.0% (11/50)	10.0% (5/50)	68.0% (34/50)	67.6% (50/74)
Ear creases/pits	17.9% (47/263)	6.8% (18/263)	75.3% (198/263)	66.8% (263/394)
Maxillary hypoplasia	29.4% (20/68)	11.8% (8/68)	58.8% (40/68)	65.4% (68/104)
Macrosomia	29.7% (80/269)	8.2% (22/269)	62.1% (167/269)	64.4% (269/418)
Neonatal hypoglycaemia	28.9% (58/201)	8.5% (17/201)	62.7% (126/201)	62.4% (201/322)
Umbilical hernia	33.8% (47/139)	10.8% (15/139)	55.4% (77/139)	59.9% (139/232)
Inguinal hernia	18.2% (4/22)	0.0% (0/22)	81.8% (18/22)	59.5% (22/37)
Congenital heart defects	18.0% (9/50)	10.0% (5/50)	72.0% (36/50)	55.6% (50/90)
Embryonal tumours	43.8% (7/16)	37.5% (6/16)	18.8% (3/16)	48.5% (16/33)
Hemihypertrophy	57.3% (98/171)	7.6% (13/171)	35.1% (60/171)	38.2% (171/448)

IC1, imprinting centre 1; IC2, imprinting centre 2; pUPD, paternal uniparental disomy.

Of those with a molecular diagnosis of BWS, 3.4% (17/507) had an embryonal tumour at referral (mean age 3.7 years, range 0 to 16 years (precise age data unavailable for five individuals)). Eight individuals had Wilms’ tumour (IC1 in four, IC2 in one, pUPD in two, and an 11p15.5 duplication in one individual), six a hepatoblastoma (IC2 in one, and pUPD in five individuals), one an adrenal cortical carcinoma (pUPD), and one a rhabdomyosarcoma (IC2). Of these, one individual had both a Wilms’ tumour and a hepatoblastoma (IC1). Interestingly, a further 17 mutation negative referrals had embryonal tumours, with the number of clinical features ranging from one to nine (median three).

### Genotype-phenotype associations

**Figure 1:**
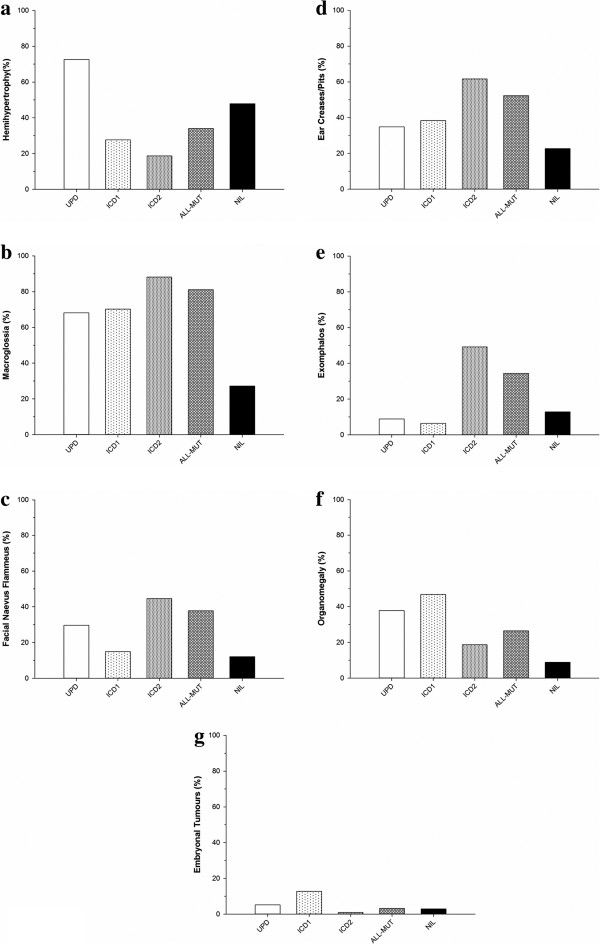
**Frequencies of Beckwith-Wiedemann syndrome clinical features according to molecular subtype. (a)** Hemihypertrophy, **(b)** macroglossia, **(c)** facial naevus flammeus, **(d)** ear creases/pits, **(e)** exomphalos, **(f)** organomegaly, and **(g)** embryonal tumours. ALL-MUT, all mutations; IC1, imprinting centre 1; IC2, imprinting centre 2; NIL, no mutations; pUPD, paternal uniparental disomy.

We compared the frequency of different clinical features of BWS between four molecular subgroups; (a) pUPD, (b) IC1 hypermethylation, (c) IC2 hypomethylation, and (d) a normal methylation profile (Figure 1). Significant intergroup differences amongst those with a positive molecular diagnosis included increased frequencies of hemihypertrophy in those with pUPD compared to the IC1 and IC2 subgroups (72.6% versus 27.7% and 18.7%; *P* < 0.001 and *P* < 0.001, respectively), macroglossia in the IC2 subgroup (88.2%) compared to pUPD (68.1%; *P* < 0.001) and IC1 (70.2%; *P* = 0.003), FNF in IC2 (44.5%) vs pUPD (29.6%; *P* = 0.003) and IC1 (14.9%; *P* < 0.001), ear creases/pits in IC2 (61.7%) vs pUPD (34.8%; *P* < 0.001) and IC1 (38.3%; *P* = 0.004), and exomphalos in IC2 (49.2%) vs pUPD (8.9%; *P* < 0.001) and IC1 (6.4%; *P* < 0.001). Conversely, there were reduced frequencies of organomegaly and embryonal tumours in the IC2 subgroup (18.7% and 0.9%, respectively) compared to pUPD (37.8%, *P* < 0.001; and 5.2%, *P* = 0.009, respectively) and IC1 (46.8%, *P* < 0.001; and 12.8%, *P* < 0.001, respectively).

Whilst there was a significant increase in the frequency of umbilical hernias in pUPD (34.8%) in comparison to IC2 (24.0%; *P* = 0.021), the differences between IC1 (31.9%) and IC2 were not statistically significant (*P* = 0.279). Similarly, for diastasis recti, increased frequencies in the IC1 subgroup (21.3%) relative to IC2 (5.6%; *P* = 0.001) were evident, but there were no significant differences between pUPD (10.4%) and IC2 (*P* = 0.074). There were no significant intergroup differences in frequencies for macrosomia, neonatal hypoglycaemia, polyhydramnios, prognathism, maxillary hypoplasia, congenital heart defects, or inguinal hernias.

### Clinical features predictive of positive molecular findings

**Figure 2:**
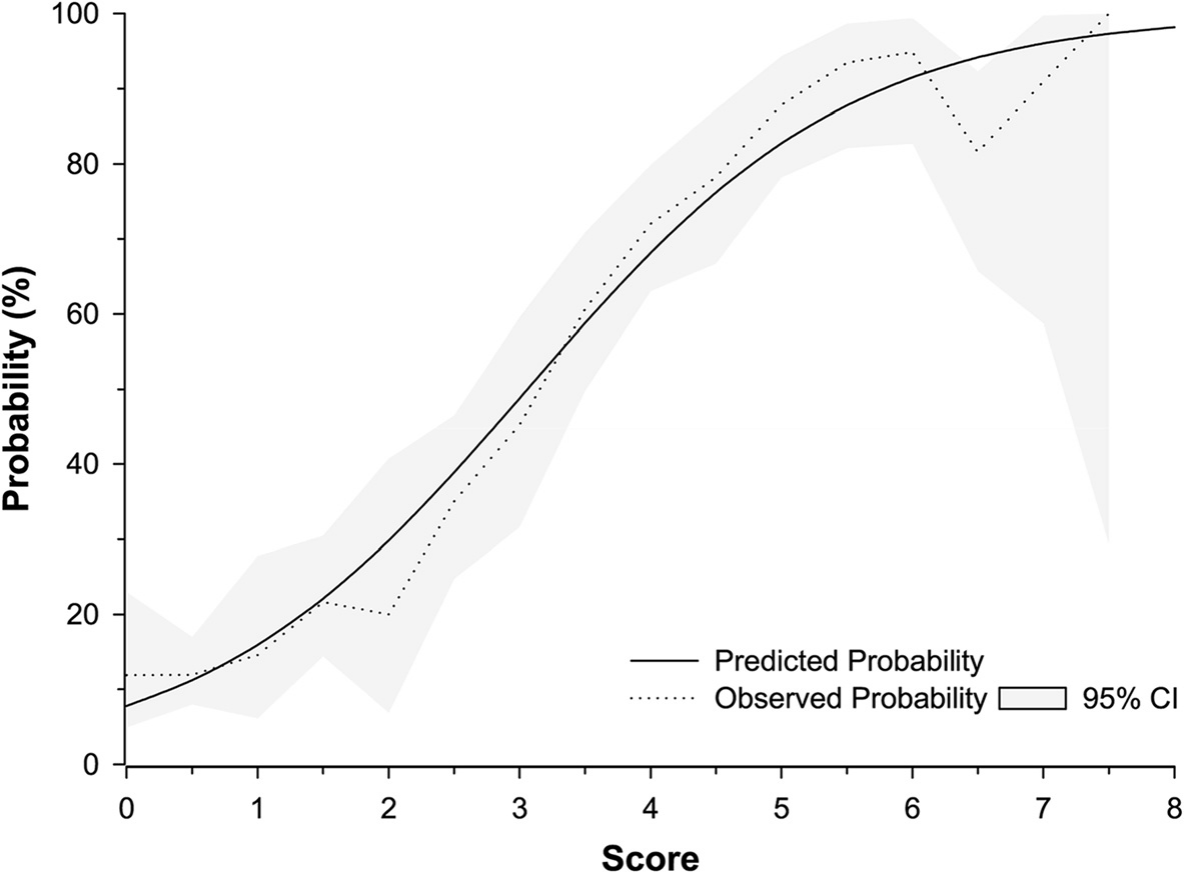
**Predicted and observed probabilities for a Beckwith-Wiedemann syndrome methylation abnormality based on the new scoring system.**$\left(\text{methylation}\;\text{abnormality}\right)=\frac{e^{‒2.47+\;0.808\left(\sum \;\text{scores}\right)}}{1\;+\;e^{‒2.47+\;0.808\left(\sum \;\text{scores}\right)}}$.

We investigated which clinical features might have the best predictive value for a positive diagnostic test for an abnormal IC1 and/or IC2 methylation. Using logistic regression, a backwards stepwise selection procedure yielded the following clinical features for inclusion in our scoring system; macroglossia, exomphalos, organomegaly, macrosomia, FNF, neonatal hypoglycaemia, and hemihypertrophy (Table 2). Since macroglossia and exomphalos had the highest regression coefficient estimates, appropriately, these were weighted with the highest scores, whilst neonatal hypoglycaemia and hemihypertrophy had the lowest (Table 3). In our new scoring system, the probability of a molecular abnormality ranges from 7.8% for a score of 0 to 98.2% for a score of 8 (Figure 2).

**Table 2:** Logistic regression analysis for the prediction of a Beckwith-Wiedemann syndrome molecular abnormality

		**Bootstrap statistics**		
	**Estimate**	**Bias-corrected estimate**	**OR (95% CI)**	***P***
Constant	−2.45	−2.47	0.08 (0.06-0.13)	<0.001
Macroglossia	2.08	2.10	8.17 (5.70-11.02)	<0.001
Exomphalos	1.14	1.15	3.16 (2.07-4.62)	<0.001
Organomegaly	0.93	0.94	2.55 (1.64-4.62)	<0.001
Macrosomia	0.78	0.79	2.19 (1.58-2.97)	<0.001
Facial naevus flammeus	0.74	0.75	2.12 (1.44-3.00)	<0.001
Hypoglycaemia	0.40	0.41	1.50 (1.06-2.08)	0.021
Hemihypertrophy	0.40	0.41	1.50 (1.04-2.14)	0.022

OR, odds ratio.

**Table 3:** Beckwith-Wiedemann syndrome molecular abnormality outcome score

	**Score**
Macroglossia	2.5
Exomphalos	1.5
Organomegaly	1
Macrosomia	1
Facial naevus flammeus	1
Hemihypertrophy	0.5
Hypoglycaemia	0.5

**Figure 3:**
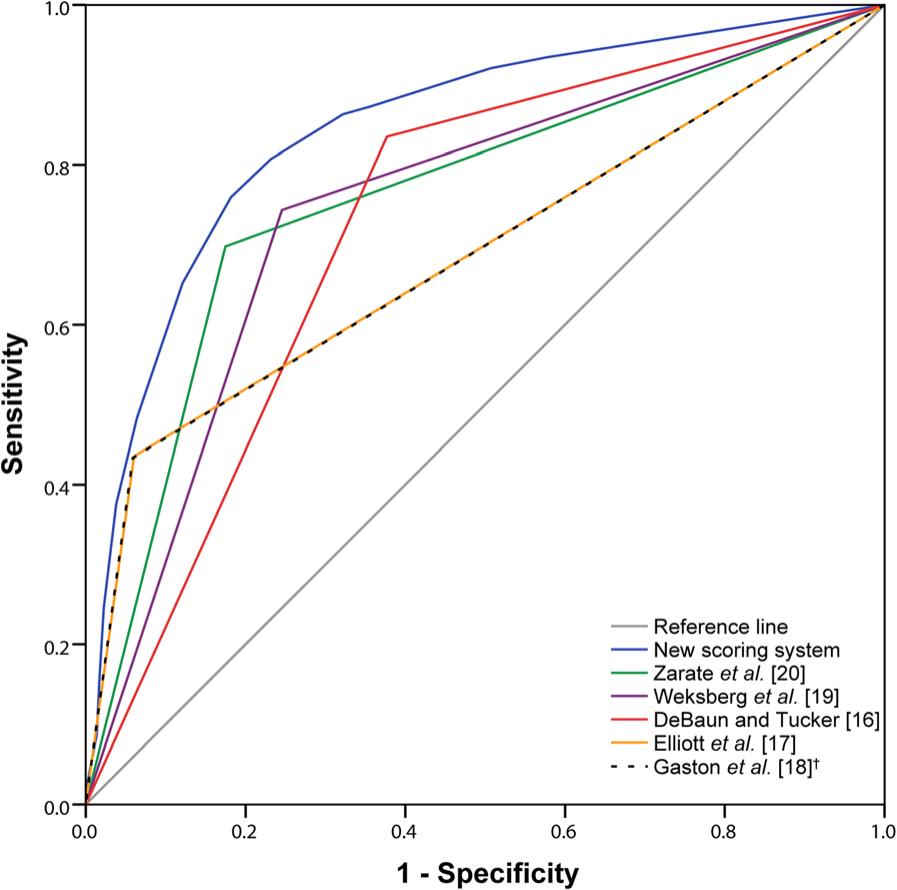
**Receiver operating characteristic curves comparing the proposed new scoring system against existing clinical diagnostic criteria for a positive Beckwith-Wiedemann syndrome methylation abnormality. **^†^Complete classification of Beckwith-Wiedemann syndrome was used (see Table 5).

In evaluating the performance of our scoring system, we used a probability threshold of 0.5 (equating to a score of 3.06) for a positive molecular diagnosis. Given that a score of 3.0 equates to a probability of 0.49, we settled for a minimum score of 3.5 (probability of 0.59). Thus, our simplified scoring system, with few and easy to distinguish features, has positive and negative predictive values of 78.4% and 79.6%, respectively (Table 4). Whilst the criteria by DeBaun and Tucker [16] had the greatest sensitivity of 83.5%, this was compounded with the lowest specificity of 62.3%. Similarly, a specificity of 94.1% by Gaston *et al.*[18] (using their complete classification of BWS) was paired with the lowest sensitivity of 43.3% in the series. Thus, whilst our new scoring system does not have the best sensitivity (75.9%) and specificity (81.8%), for any given sensitivity it has the greatest specificity, and for any given specificity the greatest sensitivity (Figure 3). Indeed, our new model has the greatest AUC of the ROC curve of 0.85 (95% CI, 0.83 to 0.87). Moreover, cross-validation of the model yielded a comparable AUC of 0.84 (95% CI, 0.81 to 0.86).

**Table 4:** Scoring outcomes of different Beckwith-Wiedemann syndrome clinical diagnostic criteria

	**Sensitivity**	**Specificity**	**Positive predictive value**	**Negative predictive value**
New scoring system^†^	75.9%	81.8%	78.4%	79.6%
Elliott *et al. *[17]	43.5%	93.9%	86.2%	65.7%
DeBaun and Tucker [16]	83.5%	62.3%	65.8%	81.3%
Weksberg *et al. *[19]	74.4%	75.4%	72.5%	77.2%
Zarate *et al.*[20]	69.8%	82.5%	77.7%	75.8%
Gaston *et al. *[18]^‡^	43.3%	94.1%	86.5%	65.6%

^†^Probability threshold of 0.5 (outcome score ≥3.5) was used for a positive molecular diagnosis. ^‡^Complete classification of Beckwith-Wiedemann syndrome was used (see Table 5).

## Discussion

Previously, we and others have reported epigenotype-phenotype associations in BWS [12,15,19,23-25]. In particular, molecular subgroups associated with CDKN1C loss of function (*CDKN1C* mutations and IC2 epimutations) have a significantly higher frequency of exomphalos than in patients with pUPD or IC1 epimutations (associated with biallelic expression of IGF2), whereas the risk of Wilms’ tumour is higher in the latter two groups. In this current study, an analysis of >500 patients with pUPD, IC1 and IC2 epimutations identified additional potential genotype-phenotype associations. Thus, isolated IC2 epimutations have a significantly increased association with macroglossia and FNF, whilst the pUPD and IC1 subgroups have a greater association with organomegaly than in IC2.

We further identified the clinical features that are most predictive of a positive molecular genetic test for abnormal methylation at the two 11p15.5 ICs. The most common molecular abnormality in BWS is loss of maternal allele methylation at IC2 (found in the 50 to 60% of cases with IC2 epimutations and in the 20% with pUPD) followed by gain of maternal allele methylation at IC1 (found in 5 to 10% of cases with IC1 epimutations and in the 20% with pUPD) [22]. IC1 and IC2 methylation profiling can also detect copy number abnormalities – most of which are IGF2/H19 duplications and large scale *CDKN1C*/IC2 deletions – though each of these findings is rare [10]. Although patients with germline *CDKN1C* mutations will not be detected, these are infrequent in sporadic cases, and only about 15% of BWS patients have a positive family history [10,15]. It should also be noted that pUPD is usually mosaic (and IC1 and IC2 epimutations commonly are) and low level mosaicism might not be detected by some methods of methylation analysis [21]. Hence, a normal ‘BWS methylation assay’ does not necessarily exclude a diagnosis of BWS - though it may be useful in prioritising further investigations. Thus a ‘BWS methylation assay’ is well established as the initial investigation of choice for potential cases and, if negative, then further investigations are generally only initiated in selected cases (for example, *CDKN1C* mutation analysis in familial cases or cases with the highly suspect clinical features of macroglossia and exomphalos) [10,25].

As genetic testing becomes less expensive and more accessible, testing is frequently requested by non-specialist clinicians, and it becomes increasingly useful to have criteria for identifying those patients who are most likely to have a positive test. Traditionally, clinical diagnostic criteria are used to select patients for molecular investigations. However, in the case of BWS clinical diagnostic criteria might be satisfied but molecular testing might be negative and, conversely, a molecular abnormality might be detected in a patient who does not satisfy clinical diagnostic criteria – such as in isolated hemihypertrophy. Here, we have focused on identifying a set of clinical criteria that are most predictive of a positive ‘BWS methylation assay’. Initially, we evaluated different sets of proposed clinical diagnostic criteria and, although some performed better than others, none was strikingly superior. We therefore used logistical regression analysis to identify those features most predictive of a positive ‘BWS methylation assay’ test and then developed a weighted clinical scoring system that was demonstrated to outperform previously reported sets of clinical diagnostic criteria [16-20]. Hence, we propose that this new scoring system should be used to guide which individuals with suspected BWS should be selected for investigation with a ‘BWS methylation assay’ test. However, it should be noted that there are limitations to this scoring system. Firstly, less frequent, but potentially significant, clinical features such as embryonal tumours are not incorporated into the scoring system because there is insufficient data to support their inclusion, and so the scoring system would not be appropriate for patients presenting with a BWS-related tumour. Secondly, the scoring system is to predict a positive result for a ‘BWS methylation assay’ and not a positive result for all BWS-associated molecular abnormalities. Thus, in familial BWS, where *CDKN1C* mutations are the most frequent abnormality [22], a scoring system designed to predict an abnormal ‘BWS methylation assay’ would not be appropriate as a normal IC1/2 methylation profile and a positive family history of maternally inherited BWS would indicate a requirement for *CDKN1C* mutation analysis [25]. Similarly, this is also indicated in sporadic cases with an abdominal wall defect (umbilical hernia or exomphalos) and no hemihypertrophy. Although our cohort was not comprehensively tested for *CDKN1C* mutations, 2.9% (17/584) of referrals with a normal ‘BWS methylation assay’ result were later found to have a *CDKN1C* mutation (mean score 4.1, range 0 to 7). Furthermore, the challenge that detection of low-level mosaicism brings to BWS diagnostics must be considered [21]. Thus, given the increased risk of tumorigenesis in individuals with isolated hemihypertrophy [26], and the difficulty in identifying a molecular abnormality, such individuals may benefit from further sensitive testing [21].

Although our scoring system focuses on improving the diagnostic rate of BWS, in comparison to current clinical diagnostic criteria, for any given sensitivity it has the highest specificity and *vice versa*, thus improving the overall diagnosis of the disorder. Moreover, in view of the tumour risk to a missed diagnosis of BWS, a molecular diagnostic or specialist genetic referral may still be warranted in individuals with borderline scores (3.0). Indeed, as testing becomes increasingly less expensive, the probability threshold for a BWS methylation abnormality can be reduced in order to improve overall diagnosis at the cost of a less-specific scoring system. Nevertheless, multicentre studies of even larger datasets will enable further refinement of our scoring system – in particular the incorporation of rarer clinical features that would increase the prior probability of BWS (for example, positive family history, history of assisted reproductive technology conception, and embryonal tumours) and also allow bespoke predictions for specific BWS molecular subtypes.

## Conclusions

In summary, we suggest that this novel tool will facilitate selection of patients with suspected BWS for routine diagnostic testing and so improve the diagnosis of the disorder.

## Methods

### Patients

We systematically collected clinical and molecular data on children and adults with suspected BWS referred to a molecular diagnostic testing service between April 2003 and October 2013. Clinical data were collected by a standardised questionnaire completed by the clinician who ordered the molecular assay and by inspecting clinical notes. Not all the clinical data requested was available, which is reflected by the variable number of clinical features. Consent for diagnostic testing was provided by the patient or by the parent/guardian for children. The collection of clinical and molecular data to evaluate the diagnostic testing service was approved by the Birmingham Women’s Hospital Research and Development Office.

### Methylation studies

DNA was isolated from peripheral blood lymphocytes or tissue samples by standard procedures. Methylation analysis was performed in the West Midlands Regional Molecular Genetics Laboratory using a methylation-sensitive multiplex ligation-dependent probe amplification (MS-MLPA) kit (SALSA MLPA kit ME030; MRC-Holland, Amsterdam, The Netherlands) and/or pyrosequencing, with both methods giving comparable results [10,27]. A categorisation of a normal or an abnormal methylation analysis assay result was made according to the clinical diagnostic report issued by the laboratory. Analysis of the MS-MLPA data was performed using the GeneMarker v1.70 (SoftGenetics, Pennsylvania, USA) software such that the ratio of normalised peak intensities was compared between the reference and the sample trace; ratios of ≤0.75 and ≥1.25 for each of the four methylation sensitive probes (four at IC1 and four at IC2) was taken to indicate loss or gain of methylation, respectively. Pyrosequencing results were analysed using the PyroMark ID (Qiagen, Venlo, The Netherlands) software (using ‘allele quantification mode’) to calculate an average methylation value for the seven CpG sites in the IC2 assay and the four CpG sites in the IC1 assay. A cohort of 89 normal controls was used to calculate the average methylation index (MI) for each of the assays, and patients with a MI greater than or less than three standard deviations from the mean of normal controls were categorised as hyper- or hypomethylated, respectively. The presence or absence of pUPD was confirmed by microsatellite analysis using markers that mapped to 11p15.5 [28]. Equivocal results were excluded from the analysis.

### Statistics

We utilised logistic regression in order to determine the predictive power for a positive methylation assay test of current BWS clinical diagnostic criteria (Table 5). We developed our own model using stepwise logistic regression for variable selection [29], with the BWS MS-MLPA result as the dependent variable. Using backwards selection, we eliminated statistically insignificant predictors to arrive at a final parsimonious model. Bootstrap resampling was performed in order to arrive at bias-corrected regression coefficients, odds ratios, and 95% CIs; 100,000 samples were drawn with replacement so as to arrive at stable estimates [30]. A weighted scoring system was devised according to the regression coefficients, and predictions of methylation abnormalities based upon the sum of the scores calculated using a simple logistic regression. Receiver operating characteristic (ROC) curves were used to determine the accuracy of our new model and current diagnostic criteria, and were quantified with respect to the area under the ROC curve (AUC). In the new model, a probability threshold of 0.5 was used for a positive molecular diagnosis. We validated our model using a 10-fold cross-validation procedure [29]. Fisher’s exact testing was used as appropriate. All tests of significance were two-sided and we used a statistical significance threshold of *P* = 0.05. Data were analysed using statistical packages R v3.0.2 (http://www.r-project.org/) and SPSS v20 (SPSS Inc., Chicago, Illinois, USA).

**Table 5:** Published clinical diagnostic criteria for Beckwith-Wiedemann syndrome

	**Elliott *et al.*** [17]	**DeBaun and Tucker**[16]	**Weksberg *et al.*** [19]	**Zarate *et al.*** [20]	**Gaston *et al.*** [18]
Major features	Abdominal wall defect	Abdominal wall defect	Abdominal wall defect	Abdominal wall defect	Abdominal wall defect
	Macroglossia	Ear creases/pits	Ear creases/pits	Macroglossia	Macroglossia
	Macrosomia	Hypoglycaemia	Embryonal tumours	Macrosomia	Macrosomia
		Macroglossia			Organomegaly
		Macrosomia	Hemihypertrophy		
			Macroglossia		
			Macrosomia		
Minor features	Ear creases/pits	Nil	Hypoglycaemia	Cardiomegaly	Ear creases/pits
	Facial naevus flammeus		Organomegaly	Ear creases/pits	Facial naevus flammeus
	Hemihypertrophy				Hemihypertrophy
	Hypoglycaemia				
					Hypoglycaemia
	Nephromegaly				
			Renal malformation	Facial naevus flammeus	
				Hemihypertrophy	
				Hypoglycaemia	
				Mid-face hypoplasia	
				Polyhydramnios	
Clinical diagnosis of Beckwith-Wiedemann syndrome	At least three major features, or two major features plus three or more minor features	At least two major features	At least three major features, or two major features and one or more minor features	At least three major features, or two major features and one or more minor features	Complete and incomplete Beckwith-Wiedemann syndrome classification.
					Complete – at least three major features.
					Incomplete – less than three major features and one or more minor features

## Abbreviations

AUC: area under the curve; BWS: Beckwith-Wiedemann syndrome; FNF: facial naevus flammeus; IC: imprinting centre; MI: methylation index; MS-MLPA: methylation-sensitive multiplex ligation-dependent probe amplification; pUPD: paternal uniparental disomy; ROC: receiver operating characteristic.

## Competing interests

The authors declare that they have no competing interests.

## Authors’ contributions

AI carried out the data analysis and co-wrote the first draft of the manuscript; GK, RPD, JB and DL provided clinical data for analysis; CH and FMacD undertook/supervised molecular epigenetic studies; PN supervised the data analysis; EM conceived and supervised the study and data analysis and co-wrote the first draft of the manuscript. All authors read, critically revised and approved the final manuscript.
